# Sorcin Activates the Brain PMCA and Blocks the Inhibitory Effects of Molecular Markers of Alzheimer’s Disease on the Pump Activity

**DOI:** 10.3390/ijms22116055

**Published:** 2021-06-03

**Authors:** Maria Berrocal, Lucia Saez, Ana M. Mata

**Affiliations:** Departamento de Bioquímica y Biología Molecular y Genética, Facultad de Ciencias and Instituto de Biomarcadores de Patologías Moleculares, Universidad de Extremadura, 06006 Badajoz, Spain; mabeca@unex.es (M.B.); lusaezm@unex.es (L.S.)

**Keywords:** sorcin, plasma membrane Ca^2+^-ATPase (PMCA), amyloid-β peptide, tau, functional modulation, neurodegeneration

## Abstract

Since dysregulation of intracellular calcium (Ca^2+^) levels is a common occurrence in neurodegenerative diseases, including Alzheimer’s disease (AD), the study of proteins that can correct neuronal Ca^2+^ dysregulation is of great interest. In previous work, we have shown that plasma membrane Ca^2+^-ATPase (PMCA), a high-affinity Ca^2+^ pump, is functionally impaired in AD and is inhibited by amyloid-β peptide (Aβ) and tau, two key components of pathological AD hallmarks. On the other hand, sorcin is a Ca^2+^-binding protein highly expressed in the brain, although its mechanism of action is far from being clear. Sorcin has been shown to interact with the intracellular sarco(endo)plasmic reticulum Ca^2+^-ATPase (SERCA), and other modulators of intracellular Ca^2+^ signaling, such as the ryanodine receptor or presenilin 2, which is closely associated with AD. The present work focuses on sorcin in search of new regulators of PMCA and antagonists of Aβ and tau toxicity. Results show sorcin as an activator of PMCA, which also prevents the inhibitory effects of Aβ and tau on the pump, and counteracts the neurotoxicity of Aβ and tau by interacting with them.

## 1. Introduction

The soluble resistance-related calcium-binding protein (sorcin) is a member of the penta EF-hand family. There are two main isoforms, the 22 kDa isoform which is the most abundant one and is found in the cytosol, and the 18 kDa isoform, which is localized in the mitochondria [1]. Mass spectrometry analysis and database searching have revealed that sorcin is widely expressed in most human tissues, such as skeletal muscle, kidney, brain, cardiac muscle, breast and skin [2,3], and it is overexpressed in a variety of human tumors and human cancer cell lines [4,5,6]. Binding of calcium ions (Ca^2+^) is essential to translocate sorcin from the cytoplasm to the cell membrane, and there it can interact with other membrane proteins involved in Ca^2+^ homeostasis [2,7]. Through the binding of Ca^2+^ to sorcin, this protein plays a significant role in cancer cells [8], and in the regulation of angiogenesis [9], mitosis [10] and endoplasmic reticulum (ER) stress [11], as revised in Colotti et al. [2] and Mao et al. [3]. The involvement of sorcin in apoptosis [12] and in metastasis of several cancer types has also been described [13,14].

Sorcin interacts and inhibits the ryanodine receptor [15] and activates the sarco(endo)plasmic reticulum Ca^2+^ ATPase isoform SERCA2a [16], thereby promoting Ca^2+^ accumulation in the ER. In the plasma membrane, sorcin modulates the L-type Ca^2+^ channels [17] and activates the Na^+^–Ca^2+^ exchangers [18]. Sorcin is also able to increase mitochondrial Ca^2+^ concentration [19].

Increasing evidences highlight the association of sorcin with neurodegeneration. In fact, it has been shown that sorcin interacts with presenilin 2, a component of the γ-secretase, which is involved in the generation of 39–42 amino acid amyloid-β peptides (Aβ) from cleavage of the Aβ protein precursor in Alzheimer’s disease (AD) [20]. Presenilins 1 and 2 have been found to form passive ER Ca^2+^ leak channels, but this function is altered by mutations associated with familial AD, independently of its γ-secretase activities [21]. Recently, it has been shown that sorcin is downregulated in the hippocampus of mice with a partial repression of AMP-activated protein kinase, a protein that seems to be hyperactive in neurodegenerative disorders [22]. These studies point to a growing interest in sorcin research concentrated on the role it may play in neurodegeneration by regulating the function of other proteins involved in Ca^2+^ signaling. Most studies have focused on the association of sorcin with ER proteins, and a recent work highlights the involvement of sorcin in ER stress in neurodegeneration [23].

In the present work, we have identified sorcin as a novel activator of the plasma membrane Ca^2+^-ATPase (PMCA), which is a crucial component of the mechanisms of Ca^2+^ efflux. This high-affinity Ca^2+^ pump plays a major role in the regulation and fine-tuning of cytosolic Ca^2+^ concentrations, by extruding Ca^2+^ out of the cell at the expense of ATP hydrolysis. Sorcin can also prevent PMCA inhibition by Aβ and tau, two key components of the pathological hallmarks of AD. Besides, sorcin is shown as a potent blocking agent of toxicity induced by Aβ and tau in human neuroblastoma cells, also counteracting their inhibitory effects on the endogenous PMCA.

## 2. Results

### 2.1. Sorcin Activates the Plasma Membrane Ca^2+^-ATPase

Previous studies by other authors have shown that sorcin activates the intracellular SERCA2a in cardiomyocites [16]. Since PMCA plays an essential role in maintaining Ca^2+^ homeostasis by removing the excess of intracellular Ca^2+^ out of the cell and is a highly regulated pump, in this work, we have analyzed the effect of sorcin on PMCA, using purified pig brain protein, which has been extensively characterized in our laboratory, as the simplest system.

**Figure 1:**
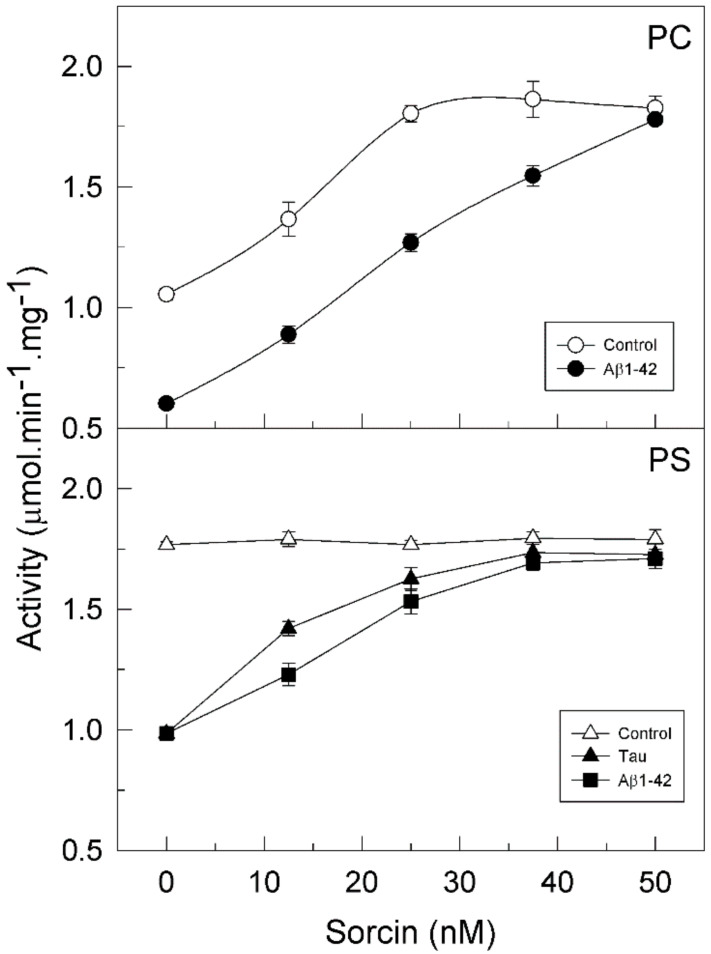
Sorcin activates the plasma membrane Ca^2+^-ATPase (PMCA) activity and prevents the inhibition of the pump by amyloid-β peptide (Aβ1-42) and tau. Purified PMCA (2.5 μg) was reconstituted with phosphatidylcholine (PC) (○) or phosphatidylserine (PS) (Δ), and incubated for 2 min at 37 °C, with Ca^2+^ and increasing concentrations of sorcin, in the absence or presence of 30 µM Aβ1-42 (●■) or 300 nM tau (▲) in 25 µL. Afterward, samples were further diluted up to 1 mL in assay medium (final concentrations of Aβ and tau were 0.75 µM and 7.5 nM, respectively). The Ca^2+^-ATPase activity was measured as described in the Methods Section. Data are mean ± SE of eight experiments performed with three preparations.

It is known that PMCA activity and its regulation depend on the ionic nature of its lipid environment. In fact, all PMCA isoforms are partially active when they are reconstituted in phosphatidylcholine (PC) and are stimulated by calmodulin (CaM), whereas they show maximal activity in phosphatidylserine (PS) and therefore are not stimulated by CaM [24,25]. Bearing this in mind, we analyzed the effect of sorcin on purified PMCA, which contains all 4 isoforms [25,26], in the presence of each lipid. The protein was incubated with increasing concentrations of sorcin (from 0.5 to 2 µM) in 25 µL, and then diluted to 1 mL in the assay medium. Therefore, the concentrations of sorcin used in the activity measurements were in the range 12.5–50 nM. Figure 1 (open circles) shows that sorcin significantly increased (about 1.8-fold) the activity of PMCA reconstituted in PC. The maximal effective concentration (EC_50_) was 12.5 nM sorcin (in the assay medium). However, the PMCA reconstituted in PS (open triangles) was already fully active and then it was not further activated by sorcin.

### 2.2. Sorcin Blocks the Inhibitory Effects of Aβ and Tau on PMCA Activity

**Figure 2:**
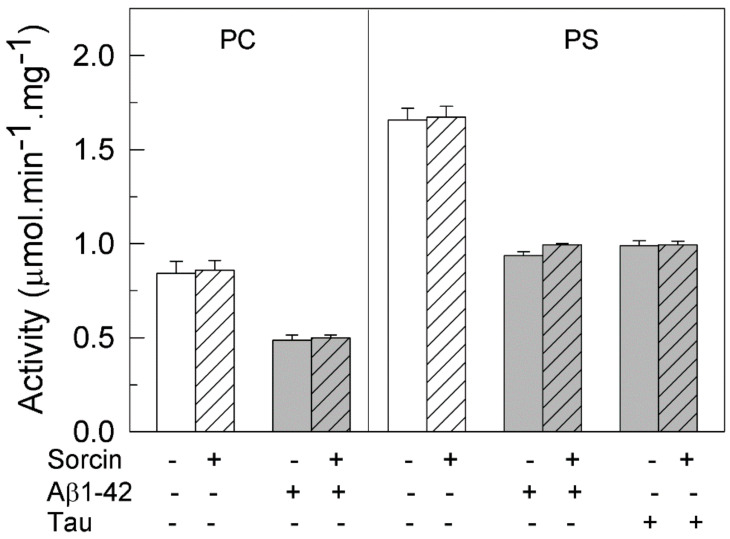
Sorcin does not to protect PMCA activity from its inhibition by Aβ and tau in the presence of EGTA. Purified pig brain PMCA (2.5 μg) reconstituted in PC or PS was treated for 2 min at 37 °C, with 2 mM EGTA, in the absence (plain bars) or presence of 1 µM sorcin (stripped bars) and 30 µM Aβ or 300 nM tau (grey bars) in 25 μL. The mixture was further diluted up to 1 mL in the assay medium (without Ca^2+^) and the reaction was started by addition of 1 mM ATP and 100 µM CaCl_2_. Data represent mean ± SE from three experiments performed in duplicate and with three different preparations.

We have previously reported that Aβ and tau, which are widely involved in AD pathology, inhibit the PMCA but not the SERCA pump, with the Aβ effect being independent of the ionic nature of the phospholipid, while tau only inhibited the PMCA activity in the presence of acidic lipids, such as PS [25]. Furthermore, we have also shown that CaM, the endogenous PMCA activator, and the phenothiazine methylene blue activated the PMCA and also prevented the inhibitory effects of both Aβ and tau on PMCA activity [25,26,27,28]. In line with those findings, we have questioned if sorcin, besides activating the PMCA, could also exert similar protective effects. Then, we have carried out kinetic assays with the purified PMCA reconstituted in neutral phospholipid PC or in acidic PS and preincubated with 30 µM Aβ or 0.3 µM tau, in the presence of increasing concentrations of sorcin and 50 µM Ca^2+^ in 25 µL. Samples were further diluted up to 1 mL in assay medium (final concentrations of Aβ and tau were 0.75 µM and 7.5 nM, respectively). As shown in Figure 1, sorcin activated the partially active PMCA, reconstituted in PC, up to the Vmax. Furthermore, sorcin decreased the inhibition produced by Aβ on pump activity in a concentration-dependent manner. As mentioned above, tau does not inhibit PMCA activity in PC and therefore the effect of sorcin on PMCA in the presence of tau is not shown. When PMCA was reconstituted in PS, it showed maximal activity, and thus it was not further activated by sorcin, although it was inhibited by Aβ and tau. Again, sorcin counteracted the inhibitory effects of Aβ or tau and blocked them completely at concentrations above 25 nM, even in the presence of both of them (result not shown). However, in the absence of Ca^2+^, 25 nM sorcin neither activated PMCA nor prevented its inhibition by Aβ and tau (Figure 2).

**Figure 3:**
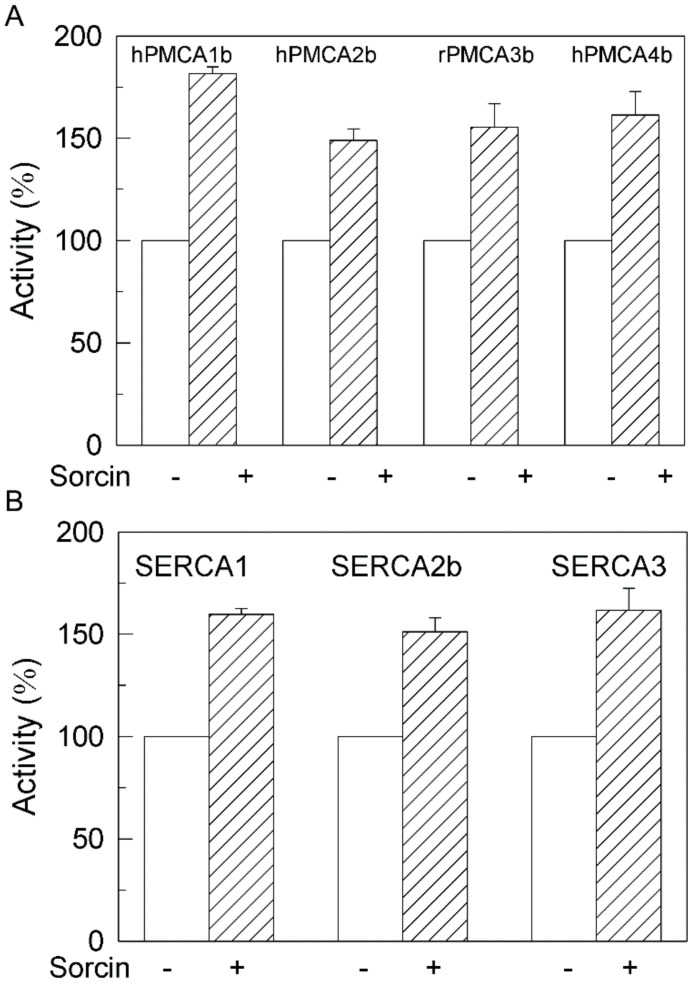
Sorcin activates all PMCA (**A**) and SERCA (**B**) isoforms. Twenty µg of membranes from COS cells overexpressing PMCA or SERCA isoforms were treated in the absence (plain bars) or presence of 1 μM sorcin, and 50 µM CaCl_2_ (stripped bars) in 25 µL, and then diluted up to 1 mL with the assay medium plus 0.01% saponin. Activity was measured as indicated in the Methods Section after addition of 1 mM ATP. The 100% activities correspond to 0.124 ± 0.003, 0.107 ± 0.004, 0.093 ± 0.006 and 0.102 ± 0.01 µmol.min^−1^.mg^−1^ for hPMCA1b, hPMCA2b, rPMCA3b and hPMCA4b respectively, and to 2.57 ± 0.018, 0.086 ± 0.015 and 0.216 ± 0.01 µmol.min^−1^.mg^−1^ for SERCA1, SERCA2b and SERCA3, respectively. Data are mean ± SE of three experiments performed in triplicate with three preparations. *p* ≤ 0.001 vs. control.

As mentioned above, the purified synaptosomal PMCA preparation is a mixture of all isoforms. In order to learn whether PMCA activation by sorcin varies with the class of isoform, we analyzed the effects of 25 nM sorcin in COS membranes overexpressing each of them. As shown in Figure 3A, sorcin activated all PMCA isoforms, between 1.5- and 1.8-fold. This activation was also observed in COS membranes overexpressing the 3 major SERCA isoforms (Figure 3B). These results indicate that sorcin is able to activate all isoforms of intracellular (SERCA) and plasma membrane (PMCA) Ca^2+^ pumps.

**Figure 4:**
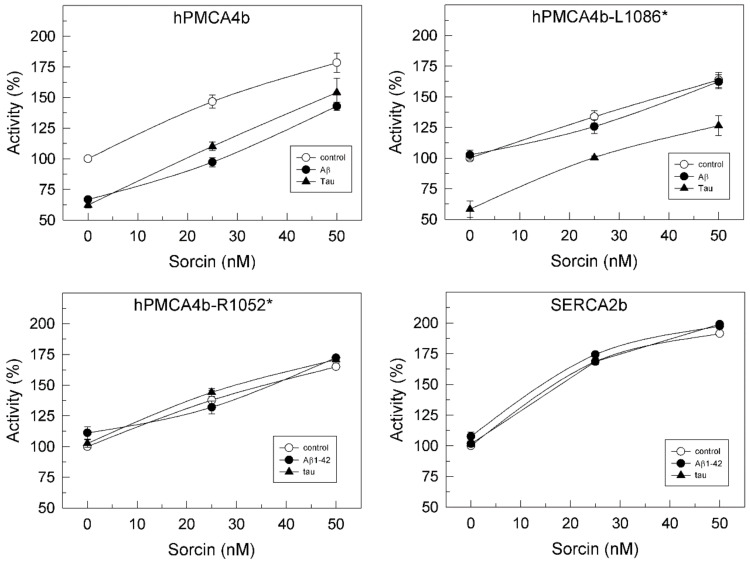
Sorcin activates Ca^2+^-ATPase activities of overexpressed full hPMCA4b, their truncated variants and SERCA2b isoforms in a concentration-dependent manner and prevents their inhibition by Aβ and tau. Twenty µg of COS cells’ membranes overexpressing full hPMCA4b, or truncated hPMCA4b-L1086* and hPMCA4b-R1052* and SERCA2b isoforms were treated with increasing concentrations of sorcin, without (○) or with 30 μM Aβ1-42 (●) or 300 nM tau (▲), in 25 µL, and then diluted up to 1 mL with the assay medium plus 0.01% saponin, resulting in the indicated final concentrations of sorcin. Activities were measured as indicated in the Methods Section, after addition of 1 mM ATP. The 100% activities correspond to 0.226 ± 0.02, 0.235 ± 0.03, 0.242 ± 0.02 and 0.190 ± 0.003 µmol. min^−1^. mg^−1^ for hPMCA4b, hPMCA-L1086*, hPMCA-L1052* and SERCA2b, respectively. Data represent mean ± SE of three experiments performed with three preparations.

We have shown in previous work that hPMCA4b is the isoform most sensitive to inhibition by Aβ [29] and tau [27]. On the other hand, SERCA2b is ubiquitously expressed and is the predominant isoform in the brain [30,31]. Therefore, we examined the effects of sorcin in the presence of Aβ and tau, on Ca^2+^-ATPase activity of COS membranes overexpressing the native hPMCA4b, truncated variants hPMCA4b-L1086* (L1086*) lacking the calmodulin binding domain (CaMBD) and hPMCA4b-R1052* (R1052*), and the SERCA2b isoform. R1052* is the PMCA variant that most closely resembles SERCA, since it lacks the whole C-terminal domain. The reason for using truncated forms of PMCA was to determine whether the binding of sorcin to PMCA occurred in the C-terminal regulatory domain of PMCA, which does not contain SERCA, or in other domains. As shown in Figure 4, the activity of native hPMCA4b measured in the absence of sorcin was inhibited by Aβ and tau, whereas the activity of L1086* was inhibited only by tau. In contrast, neither Aβ nor tau affected the activities of the shortest R1052* variant and SERCA2b, confirming our previous findings [25,26].

However, all Ca^2+^-ATPases were activated by sorcin in a concentration-dependent manner, regardless of the presence of Aβ or tau in the incubation medium. A sorcin concentration of 25 nM completely prevented the inhibition of native PMCA by 0.75 µM Aβ or 7.5 nM tau and of the truncated L1086* by 7.5 nM tau. Above that concentration, sorcin activated both native and truncated PMCAs to the same levels as those achieved in the absence of Aβ and tau. The highest percentages of Ca^2+^-ATPase activation achieved with 50 nM of sorcin were as follows: 83% ± 5% for native hPMCA4b, 63% ± 3% for L1086* and 75% for R1052*.

### 2.3. The Ca^2+^-ATPase of Human Membranes Is also Modulated by Sorcin

**Figure 5:**
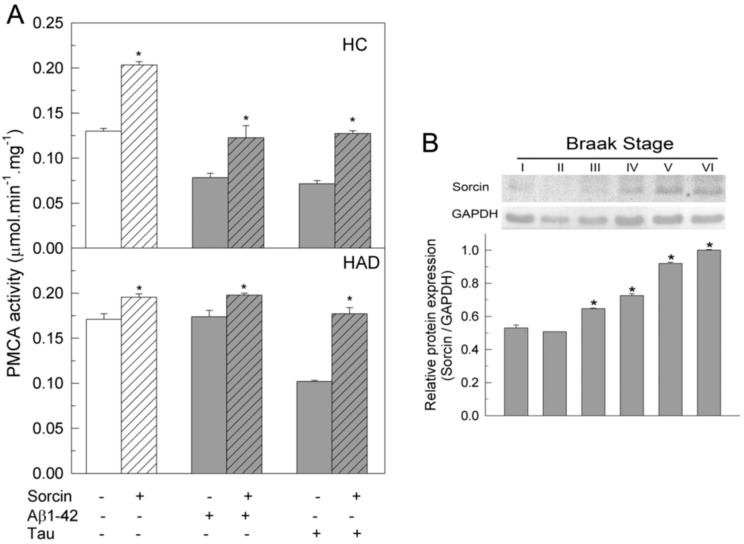
(**A**) Sorcin prevents the inhibition of PMCA activity by Aβ and/or tau in membranes from human control (HC) and AD (HAD) brain samples. Ten µg of membranes from HC (Braak stages I and II) and from HAD (Braak stages V and VI) were incubated without (plain bars) or with (stripped bars) 1 µM sorcin and 30 μM Aβ1-42 or 300 nM tau (grey bars) in 25 µL, and then diluted up to 1 mL with assay medium plus 0.01% saponin. PMCA activity was assayed after triggering the reaction with 1 mM ATP, as indicated in the Methods Section. Data represent mean ± SE values of three experiments performed with four preparations of each Braak stage. * *p* ≤ 0.001 vs. MV without sorcin. (**B**) Expression levels of sorcin in human brain samples at increasing Braak stages of AD. Twenty µg of human brain samples (SN1) of Braak stages I, II, III, IV, V and VI, were electrophoresed into 12% SDS-PAGE gel, electro-transferred to PVDF and immuno-stained with the anti-sorcin antibody, as indicated in the Methods Section. The immunoblot is representative of three assays with at least three samples from each stage. Relative expression of sorcin vs. GAPDH is shown as mean ± SE values (arbitrary units). * *p* ≤ 0.001 vs. Braak stage I.

The effects of sorcin on PMCA were also investigated in membranes prepared from human undiagnosed (HC) and diagnosed AD (HAD) brains. Samples were treated, as described above, with 1 µM sorcin and 30 µM Aβ or 0.3 µM tau, in 25 µL, and then diluted to 1 mL in assay medium. PMCA activity was measured as indicated in the Methods Section and the resulting values are depicted in Figure 5A. It was observed that in HC, the addition of sorcin increased PMCA activity by 1.75-fold, while Aβ or tau, at the concentrations assayed, produced about 50% inhibition, as expected from the results of previous work. However, the activities of membranes co-treated with sorcin and Aβ or tau were similar to those of untreated samples. These results are in agreement with those observed in purified pig brain PMCA (see Figure 1). In HAD membranes, the PMCA showed higher activity than in HC membranes, as already reported in previous studies [26,29], and it was not significantly activated by sorcin. On the other hand, Aβ did not affect the activity of HAD membranes, whereas tau inhibited PMCA by 50%, as already reported in previous studies [26,29,32], but this inhibition was prevented by co-treatment of membranes with sorcin and tau. In order to determine the expression of endogenous sorcin in human brain samples, we performed Western blots of human membranes at different Braak stages (Figure 5B). An increase in sorcin expression was observed in the later Braak stages, associated with disease progression.

### 2.4. Sorcin Prevents the Toxicity of SH-SY5Y Cells Caused by Exogenous Aβ and Tau and Preserves the Endogenous Ca^2+^-ATPase Activity

**Figure 6:**
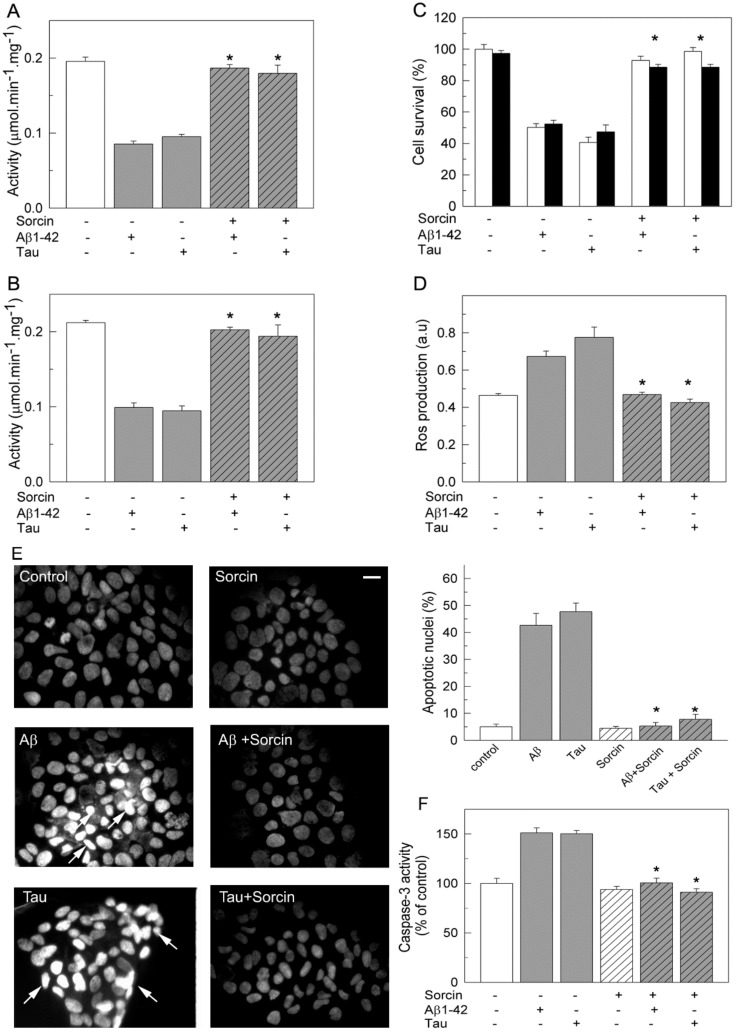
Protective effects of sorcin on Ca^2+^-ATPase activity (**A**,**B**), cell viability (**C**), ROS production (**D**) and apoptosis (**E**,**F**), in human neuroblastoma cells. SH-SY5Y cells were treated without or with 5 μM Aβ1-42 or 10 nM tau in the absence and presence of 1 μM sorcin for 24 h, as detailed in the Methods Section. (**A**) Ca^2+^-ATPase activity was assayed in 10 μg of membranes, previously treated as indicated above. (**B**) Ca^2+^-ATPase activity was measured by incubating non-treated cells with 30 μM Aβ1-42 or 300 nM tau in the absence and presence of 1 µM sorcin, in 25 µL, and then diluted up to 1 mL with assay medium plus 0.01% saponin. Activity data are mean ± SE of three independent experiments. Cells treated with Aβ or tau. (**C**) Cell viability was assayed in control and treated cells after 1 h incubation with 150 µg/mL MTT (white bars) or stained with 0.4% trypan blue (black bars). Data are expressed as percentage of untreated cells, as mean *±* SE of five independent experiments. (**D**) The production of ROS was determined by incubating non-treated and treated cells with 40 μM H_2_DCFDA, as indicated in the Methods Section. Data are expressed as mean ± SE of fluorescence intensity of three independent experiments. (**E**) DAPI staining in non-treated and treated cells. Representative fluorescent microscopy images show apoptotic cells with condensed and fragmented nuclei (white arrows). Scale bar: 10 µm. Apoptotic nuclei were quantified with respect to the total number of seeded cells. Values are mean ± SE of ten images per coverslip, obtained from three cell cultures. (**F**) Caspase-3 activity was measured in non-treated and treated cell lysates by detecting cleavage of the caspase substrate for 1 h at 37 °C. Data are represented as a percentage relative to non-treated cells (0.206 ± 0.005 fluorescence intensity/µg protein). Values are mean ± SE of three experiments. * *p* ≤ 0.001 vs. cells treated with Aβ or tau.

The protective effects of sorcin against the toxic effects of Aβ and tau were also studied in the human neuroblastoma SH-SY5Y cell line (Figure 6). Ca^2+^-ATPase activity assays (Figure 6A) showed 50% inhibition in membranes prepared from cells treated with 5 µM Aβ or 10 nM tau.

However, inhibitory effects were blocked when cells were co-treated with sorcin and Aβ or sorcin and tau, confirming the protective effect of sorcin against the inhibitory effects of Aβ and tau on PMCA activity. The Ca^2+^-ATPase activity of membranes from cells treated only with sorcin was similar to that of untreated cells (results not shown). The fact that exogenous sorcin does not stimulate Ca^2+^-ATPase activity in cells is probably due to its inability to cross the cell membrane. The same protective effects by sorcin were found when untreated cells were incubated with sorcin and Aβ or tau just before measuring the activity, indicating a direct modulation of PMCA by sorcin (Figure 6B).

In addition to kinetic assays, we evaluated the effects that co-treatments of cells with sorcin and Aβ or tau exert on cell viability. For this purpose, two cell staining methods, MTT and trypan blue (Figure 6C), were performed. Both showed that the presence of exogenous Aβ or tau in the culture medium produced a 50% decrease in cell viability, whereas co-treatments of cells with sorcin and Aβ (molar ratio 0.2:1) or sorcin and tau (molar ratio 1:1) fully protected cells from loss of viability. We also estimated the generation of intracellular reactive oxygen species (ROS) elicited by Aβ or tau in the absence and presence of sorcin using the fluorescent probe H_2_DCFDA. As shown in Figure 6D, the total ROS levels of cells treated with Aβ or tau were about 1.6–2-fold higher than those of untreated or sorcin-treated cells. However, this increase was not observed when cells were co-incubated with sorcin and Aβ or sorcin and tau. Additionally, the influence of the different treatments on the apoptosis levels of the cells was analyzed by DAPI staining (Figure 6E), showing that the number of apoptotic cells increased by 45–53% after treatments with Aβ or tau with respect to the 5–7% increase found in untreated cells or in cells treated with sorcin without or with Aβ or tau. Alternatively, apoptosis was detected by measuring caspase-3 activation (Figure 6F). As shown, Aβ and tau increased the activation of caspase-3 by 50%, while sorcin inhibited Aβ and tau effects. The protection of sorcin against the toxic effects of Aβ or tau on cell survival, ROS generation and apoptosis were also observed when the three compounds were added to the same culture (result not shown).

### 2.5. Sorcin Interacts with PMCA and also with Aβ and Tau

**Figure 7:**
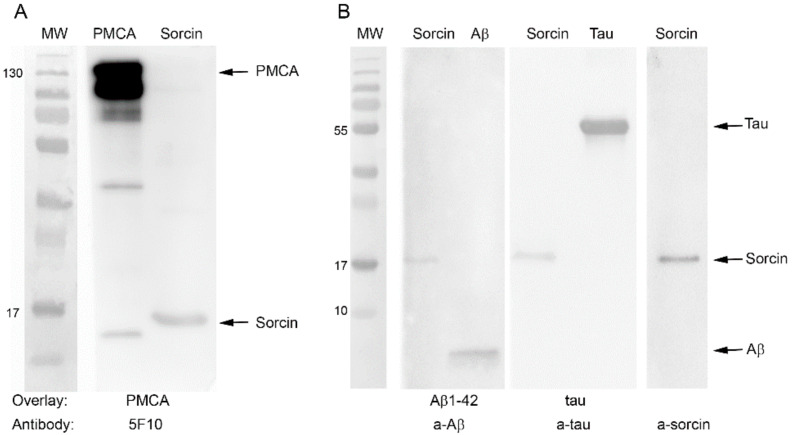
Blot overlay assays of the interaction between sorcin and PMCA, Aβ and tau. (**A**) PMCA (0.5 μg) and sorcin (1 μg) were run in 10% SDS gel, transferred to PVDF membranes and incubated sequentially with 0.5 µg PMCA and the a-PMCA antibody 5F10. The panel shows binding of PMCA to sorcin. (**B**) Sorcin (1 ug), Aβ (3 µg) and tau (3 µg) were run in three gradient 10–20% gels, transferred to PVDF and incubated sequentially with 3 µg Aβ or 3 µg tau, and with a-Aβ1-42 or a-tau antibodies, as indicated. The panel shows binding of both Aβ and tau to sorcin. It also shows a Western blot of pure sorcin with the a-sorcin antibody, as control. Immunoblots are representatives from three different assays.

The activity results obtained with purified PMCA suggest that the protective effect of sorcin against Aβ or tau may result from its direct interaction with PMCA, thus blocking the binding of Aβ or tau to the pump. However, it is possible that sorcin also interacts with both AD markers, thus preventing them from binding to PMCA. Furthermore, sorcin is unable to diffuse across the cell membrane, while extracellular Aβ or tau can be internalized in cell cultures by endocytosis [33,34,35,36,37,38], producing toxic effects [25,39]. Therefore, the results shown so far strongly suggest that the protective effect of sorcin against PMCA inhibition by Aβ and tau and also against their cytotoxicities could be due to its interaction with exogenous Aβ and tau, preventing their entries into the cells and thus their toxic effects. To analyze these possibilities in detail, we carried out overlay tests. First, we analyzed the possible sorcin–PMCA interaction, and for this, they were loaded on a 10% SDS gel, transferred to a PVDF membrane (Figure 7A) and incubated with PMCA. After several washes to remove unbound protein, the membrane was stained with the 5F10 PMCA-antibody. A representative blot showed clear binding of 5F10 antibody to the 22 kDa sorcin on the first membrane, indicating that PMCA interacts with sorcin.

The same approach was performed to study the potential interaction of sorcin with Aβ and tau (Figure 7B). In this case, sorcin and Aβ or sorcin and tau were loaded onto 10–20% SDS gels and subsequently transferred to PVDF membranes. One of them was incubated with Aβ and the other with tau, and after extensive washing, each membrane was subsequently incubated with the corresponding primary antibody, with the HRP-conjugated secondary antibody and with the chemiluminescent substrate. These blots showed that a-Aβ antibody detected a band corresponding to Aβ1-42 at the expected height of sorcin, and a-tau antibody also detected tau at the expected height of sorcin. These results indicate that sorcin interacts with both Aβ and tau. Positive controls were used to show the binding of PMCA, sorcin, Aβ and tau to their corresponding antibodies.

Additional experiments were carried out by exploring changes of the intrinsic fluorescence following excitation at 280 nm, in response to conformational changes associated with protein–protein interactions (see supplementary method). The fluorescence spectra of sorcin, PMCA, Aβ and tau were monitored before and after pre-mixing these proteins, and fluorescence values at 310 nm were obtained (Supplementary Figure S1). A quenching of fluorescence by the premixed PMCA–sorcin was observed with respect to the combined fluorescence (calculated as the sum of fluorescence peak values of PMCA and sorcin), indicating that aromatic residues on sorcin and PMCA were perturbed by the close interaction when both proteins were incubated together. Besides, the sum of individual fluorescence values of both sorcin and Aβ was higher than the fluorescence intensity of premixed sorcin–Aβ. Similarly, the sum of individual values of sorcin and tau was higher than the fluorescence intensity of the premixed sorcin–tau.

## 3. Discussion

As mentioned above, sorcin is widely distributed in human tissues. Several studies propose its involvement in Ca^2+^ homeostasis, cell cycle regulation and vesicle trafficking, diabetes, infertility and nervous system pathologies (reviewed in [40]), although most studies on sorcin are related to its overexpression in tumor cells [2,40,41] and to its regulatory role in excitation–contraction coupling in the heart through its interaction with several proteins, such as the SERCA pump [16], the ryanodine receptor intracellular Ca^2+^ channel [15,42], the Na^+^-Ca^2+^ exchanger [18] and L-type Ca^2+^ channels [43]. Mass spectrometric and database analyses have shown that sorcin is highly expressed in the brain [44,45,46]. However, its role is far from being known.

It has been shown that sorcin co-localizes with several isoforms such as the ryanodine receptor in rat and mouse brain [23,47] and with a region of presenilin-2 (a protein which is mostly associated to familial AD) in human brain tissues [20]. Few studies have identified the connection of sorcin to several proteins involved in Parkinson’s Disease and other Lewy body diseases, such as α-synuclein and synplilin-1, using phage display [48] and gene co-expression analysis [49]. Besides, a recent work [23] reported an increase in sorcin expression in neuroblastoma cells treated with a neurotoxin widely used in animal models of Parkinson’s Disease, and also in a mouse model of Huntington’s disease and in Down Syndrome human brain without or with diagnosis of AD (at ages under and over 40, respectively). The study was focused on the interaction of sorcin with ER proteins involved in Ca^2+^ signaling, such as ryanodine and Sigma-1 receptors. In the present work, we have identified sorcin as a novel activator of the high-affinity PMCA pump. Another interesting finding is that sorcin counteracts the inhibitory effects that two key components of Alzheimer’s pathology, such as Aβ and tau, exert on PMCA. Furthermore, sorcin is shown to be a potent blocker of Aβ- and tau-induced toxicity in human neuroblastoma cells, also preventing their inhibitory effects on the endogenous PMCA activity.

To our knowledge, this is the first study to show the functional effects of sorcin on the plasma membrane Ca^2+^ pump from human and pig brain and also from neuroblastoma cells. This pump is highly regulated, and is the only Ca^2+^-transport ATPase which is activated by CaM [50]. The pig brain PMCA purified by CaM-affinity chromatography is eluted in a delipidated state, and therefore lacks its activity. To activate the pump, it is necessary to reconstitute it with lipids [24]. The lipid nature is a key factor in the function of membrane proteins. We and other authors have shown that in the presence of PC, the protein is partially active and can be fully activated by CaM, whereas in the presence of PS, the PMCA undergoes a conformational change that renders it fully active, and therefore it is not further stimulated by CaM [24,50,51,52]. On the other hand, the surrounding lipids also affect the distribution of PMCA isoforms in lipid rafts microdomains [53,54,55] and their interactions with Aβ [29] and tau [25].

Sorcin activated the PMCA only when it was reconstituted in PC (since in this case it was partially active), reaching the same Vmax as in PS. Moreover, this activation was not observed in the absence of Ca^2+^, which is in agreement with the fact that sorcin interacts and regulates a variety of proteins in a Ca^2+^-dependent manner [2,56]. These effects were similar to those produced by CaM, the endogenous activator of the pump, as described above. It is well-known that at rest, PMCA is partially autoinhibited by interaction of its C-terminal CaMBD with two cytosolic loops, and that Ca^2+^-CaM activates the pump through its binding to the autoinhibitory CaMBD domain [28,57,58,59]. Given the similarity in the ways of PMCA activation by sorcin and CaM, we might think that sorcin would activate PMCA by binding to the same domain as CaM. However, in this work, we have shown that sorcin activates PMCA mutants L1086* and R1052*, lacking the CaMBD and the whole C-terminal tail respectively, and it also activates all intracellular SERCA isoforms, which do not contain this domain, whereas CaM does not affect either SERCA or the secretory pathway Ca^2+^-ATPase SPCA, which does not have the CaMBD either. Therefore, these results indicate that sorcin does not bind to the CaMBD. Then, sorcin and CaM may activate PMCA by binding to different sites on the pump. Overlay binding assays for sorcin–PMCA and PMCA–sorcin have shown interactions between both proteins. The fact that sorcin activates truncated forms of PMCA, which structurally resemble the SERCA pump, suggests that its activating effect is exerted by binding to common sites on both types of Ca^2+^-ATPases.

Of particular interest is the fact that sorcin counteracts the inhibitory effects of Aβ and tau on PMCA activity, and even blocks them completely. These results suggest that sorcin could also bind to both Aβ and tau. Overlay binding experiments confirmed the interaction of sorcin with both of them (as antibodies against Aβ and tau detected binding of the peptide and tau to sorcin transferred to PVDF). Sorcin, again, behaved like CaM with respect to its blocking effects on both inhibitors of the pump, since CaM was also able to block and even completely reverse the inhibitory effects of Aβ [28] and tau [25].

Previous studies using native and truncated hPMCA4b isoforms suggested that Aβ binds to the CaMBD of PMCA [28], while tau binding may be located at a site close to PMCA transmembrane domain 10 [25]. We have also shown, by using fluorescence experiments, that CaM has a very high affinity (Kd ~ 1 nM) for Aβ [60].

The fact that sorcin activates all PMCA and SERCA isoforms while CaM only activates PMCA indicates that sorcin does not bind to the CaMBD (which is lacking in SERCA). This is corroborated by the ability of sorcin to activate not only the native protein but also the truncated L1086* and R1052* isoforms, lacking the CaMBD and the whole C-terminal tail, respectively.

The activating effect of sorcin, at the half maximal effective concentration (EC_50_), was also seen in human brain membranes from control cases and to a lesser degree in AD, most probably because the PMCA activity is already higher in AD than in control membranes [29]. This observation raises the possibility that sorcin may play a role as an endogenous activator of the pump. In fact, Western blot assays performed in this work with membranes from control and AD human tissues revealed an increase in sorcin expression with AD progression, being more significant in the latest Braak stages, which could be associated with the higher activation of PMCA in AD samples. In addition, other authors have shown, by quantitative proteomic analysis and database searching [23,44,45,46], an increased expression of sorcin in AD-affected brain samples compared to non-AD controls.

In human tissue membranes, sorcin also prevented the inhibition of PMCA activity by Aβ and tau. From this point of view, sorcin may play a beneficial role in the function of PMCA, because, on the one hand, by activating PMCA, sorcin may help to reduce the increase in the levels of neuronal resting Ca^2+^ in AD. On the other hand, sorcin counteracts the toxic effects of Aβ and tau, thus preventing the protein from losing its function as a high-affinity Ca^2+^ transporter.

In human neuroblastoma SH-SY5Y cells, sorcin also protected PMCA from its inhibition by exogenous Aβ and tau. Sorcin is not able to cross the cell membrane, and therefore, it cannot activate endogenous PMCA in SH-SY5Y membranes. However, it has been shown that exogenous Aβ can be internalized in HEK-293 cells overexpressing APP [61,62], in neurons from cultured hippocampal slices [63,64], in mouse neuroblastoma (N2A) cells [33], and in human SH-SY5H cells [65], as reviewed in [34]. Extracellular tau has also been reported to be internalized by neurons in culture [35,36,37,38,66]. Through their internalization, Aβ and tau promote toxic effects [65,66]. Therefore, the inhibition of endogenous PMCA observed in membranes treated with exogenous Aβ or tau indicates that both of them cross the plasma membrane and inhibit PMCA. Sorcin, however, prevented those inhibitions, most likely by its interaction with exogenous Aβ and/or tau, preventing their entry into the cells. Thus, sorcin binding to Aβ and tau would result in decreased levels of free Aβ and tau crossing the membrane and inhibiting PMCA. Overlay binding and intrinsic fluorescence experiments prove that sorcin interacts with Aβ and tau. Other authors have demonstrated the interaction of sorcin with presenilin 2, a protein directly related to AD as it is an enzymatic component of the γ-secretase complex that cleaves amyloid β-protein precursor [20], and also with tau in HEK293 and SH-SY5Y cells [67]. Considering the key role of PMCA in the control of intracellular Ca^2+^ levels, and that Ca^2+^ overload is a characteristic feature of AD and other neurodegenerative diseases, the interaction of sorcin with PMCA may be a beneficial mechanism to remove the excess of cytosolic Ca^2+^ and then to restore Ca^2+^ homeostasis.

The interaction of sorcin with exogenous Aβ and with tau also explains the positive effects of sorcin observed in viability and apoptosis assays, as well as the lack of PMCA inhibition observed in human neuroblastoma SH-SY5Y cell membranes after co-treatment with sorcin and Aβ or sorcin and tau. Furthermore, exposure of cells to Aβ and tau induced an increase in ROS generation, which in turn could enhance the inhibition of PMCA activity observed in these cells, as PMCA is particularly susceptible to ROS-mediated damage [68,69].

Results of this work lead us to propose PMCA as a novel sorcin-binding protein and also to point out the interplay between sorcin and AD based on its interaction with Aβ and tau. Functional inhibition of neuronal PMCA by Aβ and tau is most probably a contributing factor to cytosolic Ca^2+^ dysregulation associated with AD. It is therefore of great interest to find compounds that prevent PMCA inhibition and thus contribute to restoring its central role in the control of cytosolic Ca^2+^. The interaction of sorcin with Aβ and tau represents a protective mechanism to prevent dysfunction of PMCA and also to reduce the severity of neurodegeneration in AD and other neurodegenerative diseases involving toxicity by Aβ and tau.

**Figure 8:**
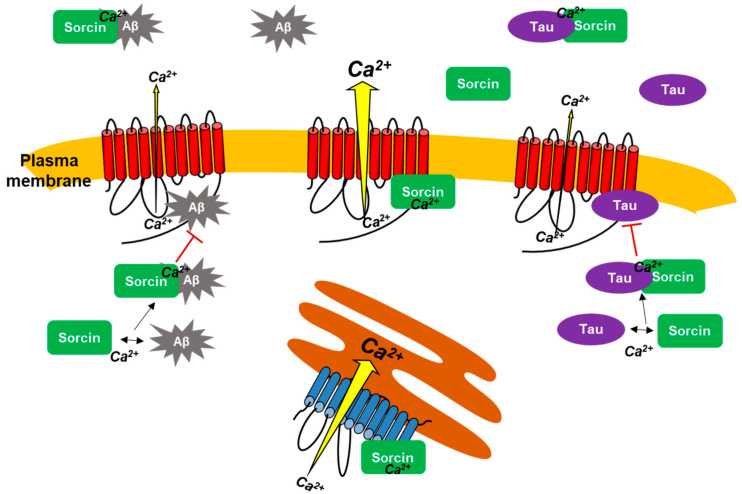
The scheme summarizes the interactions of sorcin with PMCA (red) and SERCA (blue), in the presence of Ca^2+^ that lead to Ca^2+^-ATPase activation. Sorcin also interacts with AD markers, such as tau and Aβ, preventing their binding to PMCA and their inhibitory effects on PMCA activity.

Figure 8 summarizes all interactions of sorcin with PMCA, Aβ and tau, and with SERCA described in this study by using kinetic measurements, intrinsic fluorescence, overlay binding experiments and cell cultures. Overall, this work highlighted that sorcin (1) activates the PMCA by its binding to it, and (2) interacts with two key molecular markers of AD, such as Aβ and tau, blocking their inhibitory effects on PMCA. In vitro assays suggested that the presence of exogenous sorcin may be relevant to prevent neurotoxicity of exogenous Aβ and tau with respect to cell viability and to PMCA inhibition.

## 4. Materials and Methods

Recombinant Human SRI (sorcin) was obtained from Sino Biological US Inc, Eschborn, Germany. The Aβ1-42 peptide was supplied by StabVida, Caparica, Portugal as a lyophilized powder. A 4 mg/mL stock solution was prepared by solubilization of the peptide in 1% NH_4_OH and further dilution with 100 mM HEPES/KOH (pH 7.4). All experiments were performed with Aβ1-42 previously incubated for 2 h at 37 °C [28]. Full-length tau 441 was obtained from Enzo Life Sciences, Barcelona, Spain. Brains (60–80 g) from 5-month-old pigs were obtained from a local slaughterhouse, immediately placed on ice, in 10 mM HEPES/KOH, pH 7.4, 0.32 M sucrose, 0.5 mM MgSO4, 0.1 mM PMSF and 2 mM 2-mercaptoethanol (buffer I), for transport and used within 3 h after the animals were slaughtered in order to keep cell viability and protein quality. Non-AD and AD human brain tissues of Braak stages I, II, III, IV, V and VI, ages 72–90 (5–6 g pieces of middle frontal gyrus as starting material) were provided by the Netherlands Brain Bank (NBB), Amsterdam, The Netherlands. Procedures, information and consent forms of the NBB were approved by the Medical Ethics Committee of the Vrije Universiteit Amsterdam Medical Centre on 30 April 2009. The project identification code is “Avila (537)”. Research involving human tissues was carried out following the rules of the Declaration of Helsinki of 1975 (https://www.wma.net/what-we-do/medical-ethics/declaration-of-helsinki/, accessed on 1 August 1975), revised in 2013. Research involving animal and human post-mortem tissues was approved by the ethics committee of the University of Extremadura (Ref. 66/2017). PC type XI-E from egg yolk, PS and calmodulin-agarose were obtained from Sigma. The following antibodies were used: monoclonal anti-PMCA 5F10 (Thermo Scientific, Madrid, Spain), anti-sorcin, anti-tau (H150) and anti-GAPDH antibodies (Santa Cruz Biotechnologies, Heidelberg, Germany), and the polyclonal Aβ1-42 that was raised in our laboratory against the aged Aβ1-42. This antibody was obtained by immunization of a New Zealand rabbit with a solution containing 500 μg of aged Aβ1-42 in 0.5 mL PBS and 0.5 mL Freud’s complete adjuvant. Six subsequent immunizations were performed until highest antibody levels were obtained. After the last immunization, the animal was bled, and the blood was allowed to clot for 4 h at room temperature. It was then centrifuged at 2500× *g* for 15 min at 4 °C, obtaining the polyclonal serum in the supernatant. All the material was divided into aliquots and stored at −80 °C until use.

### 4.1. Preparation of Purified Plasma Membrane Ca^2+^-ATPase from Pig Brain

The PMCA from pig brain was purified as described by Salvador and Mata [24]. Briefly, fresh pig brain (60–80 g) was cut into small pieces and homogenized in buffer I. After two centrifugations at low and high speed, the resulting pellet was resuspended in buffer I and applied to a discontinuous sucrose gradient, to isolate synatosomes. This fraction was subjected to lysis in 10 mM HEPES/KOH, pH 7.4, 1 mM EDTA and 2 mM 2-mercaptoethanol to obtain synaptic plasma membrane vesicles that were resuspended, at 6 mg/mL, in 20 mM HEPES/KOH, pH 7.4, 130 mM KCl, 0.5 mM MgCl_2_, 50 µM CaCl_2_, 15% (*w*/*v*) glycerol and 2 mM 2-mercaptoethanol, and solubilized in 0.6% (*w*/*v*) Triton X-100. The solubilized proteins were loaded onto a CaM affinity column. The purified PMCA was eluted from the column in a buffer containing 20 mM HEPES/KOH, pH 7.4, 130 mM KCl, 1 mM MgCl_2_, 2mM EDTA, 0.06% Triton X-100, 2 mM 2-mercaptoethanol and 15% glycerol, and frozen at −80 °C in small aliquots. The Bradford protein assay [70] was used to measure total protein concentration.

### 4.2. Preparation of Membrane Extracts from Cells and Human Brain Tissues

Full-length hPMCA1b, hPMCA2b, rPMCA3b and hPMCA4b isoforms and truncated variants hPMCA4b-L1086* (lacking the CaMBD) and hPMCA4b-R1052* (lacking the CaMBD and the putative ethanol binding site) [71], and pSERCA2b and hSERCA3 isoforms were expressed in fibroblast-like monkey COS-7 cells (ATCC, Rockville, MD, USA), as described by Berrocal et al. [25,28,29]. The SERCA1, prepared from rabbit sarcoplasmic reticulum, was kindly supplied by Fernando Henao (University of Extremadura, Badajoz, Spain). Membrane extracts (MV) were prepared as described by Sepulveda et al. [72]. Briefly, human brain tissues (around 2 g of starting material) were excised, and cells were scraped and pelleted. After homogenization in 10 mM HEPES/KOH, pH 7.4, 0.32 M sucrose, 0.5 mM MgSO_4_, 0.1 mM phenylmethanesulfonyl fluoride, 2 mM 2-mercaptoethanol and protease inhibitor cocktail solution (Roche Diagnostics), and centrifugation for 10 min at 1500× *g*, the supernatants (SN1) were centrifuged for 45 min at 100,000× *g*. The pellet (MV) was resuspended in 10 mM HEPES/KOH, pH 7.4, 0.32 M sucrose and stored at −80 °C until use in small aliquots. The protein concentration was determined by the Bradford method [70].

### 4.3. Ca^2+^-ATPase Activity Assays

The Ca^2+^-ATPase activity was measured with a coupled enzyme assay, following the absorbance decrease of NADH at 340 nm, as described by Salvador and Mata [24] and using pig brain delipidated purified PMCA, previously reconstituted in phospholipids or MV from cells or human brain tissues. Reconstitution of PMCA was carried out as follows: the phospholipid was prepared in chloroform:methanol (7:3) as a 10 mg/mL stock solution and was applied into a test tube and dried under N_2_ atmosphere. Then, it was dissolved in purified delipidated PMCA (2.5 µg), containing 0.06% Triton X-100, at a lipid/protein ratio of 5.3:1 (*w*/*w*) until clarity, incubated for 2 min at 37 °C without or with sorcin (at different concentrations), and/or 30 µM Aβ or 300 nM tau in 25 µL, and then diluted up to 1 mL with assay medium (50 mM HEPES/KOH, pH 7.4, 100 mM KCl, 2 mM MgCl_2_, 5 mM Na_3_N, 3.16 µM free Ca^2+^ (pCa 5.5), 0.22 mM NADH, 0.42 mM phosphoenolpyruvate, 10 IU pyruvate kinase and 28 IU lactate dehydrogenase). A final 2 min incubation was performed before triggering the reaction with 1 mM ATP. Sorcin was incubated in the presence of 50 µM CaCl_2_ or 2 mM EGTA when indicated. For the measurement of MV activity, the medium was supplemented with 0.01% saponin to allow access of substrates across plasma and intracellular membranes. Activities of MV (20 µg) from COS-7 cells overexpressing PMCA and SERCA isoforms, and from SH-SY5Y cells, were assayed by starting the reaction with 1 mM ATP, followed by addition of 3 mM EGTA (to measure Mg^2+^-ATPase activity). The SERCA1 activity was measured by using the same assay as described by Mall et al. [73], in the presence of Ca^2+^ ionophore calcimycin (A23187). The PMCA activity in human brain MV was measured as described by Sepulveda et al. [74]. Briefly, the reaction was started with 1 mM ATP and activities were determined after successive additions of 100 nM thapsigargin (to inhibit SERCA activity), 2 μM vanadate (to selectively inhibit PMCA activity) and 3 mM EGTA (to measure Mg^2+^-ATPase activity).

### 4.4. Western Blotting

Proteins from SN1 fractions (20 µg) from human brain tissue were separated by electrophoresis in 12% (*w*/*v*) SDS-polyacrylamide gels, according to the method of Laemmli [75], and electro-transferred to PVDF membranes. After blocking in Tris-buffered saline (TBS) containing 5% (*w*/*v*) of low-fat milk for 1 h, immunostaining reactions were performed by incubating the membranes overnight at 4 °C with the anti-sorcin primary antibody (1:200) diluted in TBS-0.05% (*v*/*v*)-Tween 20. Afterwards, membranes were incubated for 1 h at room temperature with peroxidase-conjugated secondary antibodies (1:3000) and developed with ECL substrate. The monoclonal anti-GAPDH antibody (1:3000) was used as a loading control protein. After extensive washing, ECL substrate was applied to the membranes and signals were visualized with a Chemidoc™ XRS+ Imaging System and quantified with Image Lab™ software 3.0.

### 4.5. Neuroblastoma Cell Cultures and Treatments

SH-SY5Y human neuroblastoma cells (Sigma-Aldrich) were grown in Dulbecco’s modified Eagle’s medium (DMEM) supplemented with 2 mM L-glutamine, 100 mU/mL penicillin, 0.1 mg/mL streptomycin and 10% heat-inactivated fetal bovine serum (FBS). Cells were incubated at 37 °C in humidified atmospheric air containing 5% CO_2_. The next day, cells were treated with 10 nM tau, 5 µM Aβ1-42 and/or 1 µM sorcin for 24 h. Afterwards, MV were prepared from SH-SY5Y to measure the Ca^2+^-ATPase activity, as detailed above.

### 4.6. Cell Viability Assay

SH-SY5Y cells were seeded overnight at a density of 10,000 cells/well in 96-well cell culture plates. Then, 5 μM Aβ1-42, 10 nM tau and/or 10 nM and 1 μM sorcin were added to the cells for 24 h. Cell viability was determined by the MTT assay according to [76]. Briefly, cells were incubated with 150 µg/mL 3-(4,5-dimethylthiazol-2-yl)-2,5-diphenyltetrazolium bromide (MTT) in phosphate-buffered-saline (PBS) for 1 h at 37 °C. Afterwards, the media was removed and 100 μL of DMSO was added to each well. MTT was reduced to a formazan precipitate by metabolically active cells, that was solubilized with DMSO and quantified at 490 and 650 nm (background subtraction) in a Varioskan Flash fluorescence spectrophotometer (Thermo Scientific). Cell viability was also measured by the trypan blue exclusion test described by Strober [77]. In brief, the media was removed, and cells were resuspended in 50 μL of PBS. The same volume of 0.4% trypan blue dye was added to the cell suspension and incubated for 3 min. Finally, 10 μL of mixture was applied to a hemacytometer and unstained (viable) cells and stained (non-viable) cells were counted. Trypan blue cell viability (%) = (total viable cells (unstained)/total cells (stained and unstained)) × 100.

### 4.7. Reactive Oxygen Species Assay

The generation of intracellular reactive oxygen species (ROS) was detected by using the fluorogenic dye 2′7′-dichlorodihydrofluorescein diacetate (H_2_DCFDA) (Invitrogen, Thermo Fisher Scientific, Madrid, Spain). SH-SY5Y cells were seeded overnight at a density of 10,000 cells/well in 96-well cell cultured plates and then treated with 5 μM Aβ1-42, 10 nM tau and/or 10 nM and 1 μM sorcin for 24 h. Afterwards, the medium was removed and 40 µM H_2_DCFDA in PBS was added to cells and incubated for 30 min at 37 °C with 5% CO_2_. The H_2_DCFDA diffuses into cell and is deacetylated by cellular esterases resulting in a non-fluorescent compound, which is oxidized by ROS into the fluorescent 2′7′-dichlorodihydrofluorescein (H_2_DCF). After washing with PBS, cell were lysed with 25 µL NaOH 1 M and PBS was added up to 200 µL final volume. The fluorescent intensity of cell extracts was measured in a Varioskan Flash fluorescence spectrophotometer (λexc = 485 nm, λem = 530 nm) [78].

### 4.8. Quantification of Apoptotic Cells

Apoptosis detection and quantification was assessed using DAPI staining and caspase-3 activity, in SH-SY5Y cells seeded overnight at a density of 60,000/well in 24-well plate and then treated with 5 μM Aβ1-42, 10 nM tau and/or 1 μM sorcin for 24 h. For DAPI staining, cells were fixed and incubated with 0.3 µM of 4′6-diamidino-2′-phenylindole dihydrochloride (DAPI) fluorescent dye to stain cell nuclei. Non-apoptotic cells presented a homogeneous distribution of DNA in a normal size, while apoptotic cells showed condensed chromatin [79,80]. The caspase-3 assay was performed as described in [81]. The caspase-3 activity was measured from the cleavage of the caspase substrate *N*-Acetyl-Asp-Glu-Val-Asp-7-amido-4-methylcoumarin in apoptotic cells, using a Varioskan Flash fluorescence spectrophotometer (λexc = 360 nm, λem = 460 nm), and data were calculated as fluorescence intensity/µg protein and represented as percentage relative to the control.

### 4.9. Overlay Binding Assay

Purified PMCA (0.5 µg) and human recombinant sorcin (1 µg) were subjected to a 10% SDS-PAGE. On the other hand, human recombinant sorcin (1 µg), tau (3 µg) and Aβ (3 µg) were subjected to a 10–20% gradient SDS-PAGE. Gels were transferred to PVDF membranes (Mb:PMCA–sorcin, Mb:sorcin–Aβ, Mb:sorcin–tau) and blocked with PBS-1% Tween 20 (PBS-T), containing 5% (*w*/*v*) low-fat milk for 1 h at room temperature. The Mb:PMCA–sorcin was incubated for 3 h at 37 °C with 0.5 µg PMCA in PBS-T. Membranes Mb:sorcin–Aβ and Mb:sorcin–tau were incubated with 3 µg Aβ and 3 µg tau respectively, and after extensive washing steps with PBS-T to remove unbound proteins, the membranes Mb:PMCA–sorcin, Mb:sorcin–Aβ and Mb:sorcin–tau were incubated with anti-PMCA (5F10), anti-Aβ and anti-tau (H150) antibodies respectively, in PBS-T o/n at 4 °C. Immunoreactivity was detected with anti-mouse or rabbit peroxidase-conjugates secondary antibodies. After washing, the membranes were developed with ECL substrate and the signal was visualized with a ChemiDoc™ XRS+ Imaging System.

### 4.10. Statistical Analysis

Data were fitted to the appropriate equation using the SigmaPlot v10 software (SPSS Inc, Chicago, IL, USA). Significant differences were determined by an unpaired Student’s *t*-test. Statistical significance was accepted for *p* ≤ 0.001.

## Data Availability

Not applicable.

## Supplementary Materials

The following are available online at https://www.mdpi.com/article/10.3390/ijms22116055/s1.

## Abbreviations

Sorcin	Soluble resistance-related calcium-binding protein
AD	Alzheimer’s Disease
PMCA	Plasma membrane Ca^2+^-ATPase
SERCA	Sarco(endo)plasmic reticulum Ca^2+^-ATPase
Aβ	Amyloid-β peptide
CaM	Calmodulin
CaMBD	Calmodulin-Binding Domain
PS	Phosphatidylserine
MV	Membrane extracts
ROS	Reactive oxygen species
PC	Phosphatidylcholine
