# Supplementary material for: Sorcin Activates the Brain PMCA and Blocks the Inhibitory Effects of Molecular Markers of Alzheimer’s Disease on the Pump Activity
Source: Int J Mol Sci. 2021 Jun 3;22(11):6055. doi: 10.3390/ijms22116055 (PMC8200006; doi:10.3390/ijms22116055)
Supplement: Supplementary file 1 [file ijms-22-06055-s001.zip › ijms-1212985-supplementary.pdf]

International Journal of
Molecular Sciences

MDPI

Supplementary Materials

Sorcin Activates the Brain PMCA and Blocks the Inhibitory Effects of Molecular Markers of Alzheimer's Disease on the Pump Activity

Maria Berrocal, Lucia Saez and Ana M. Mata *

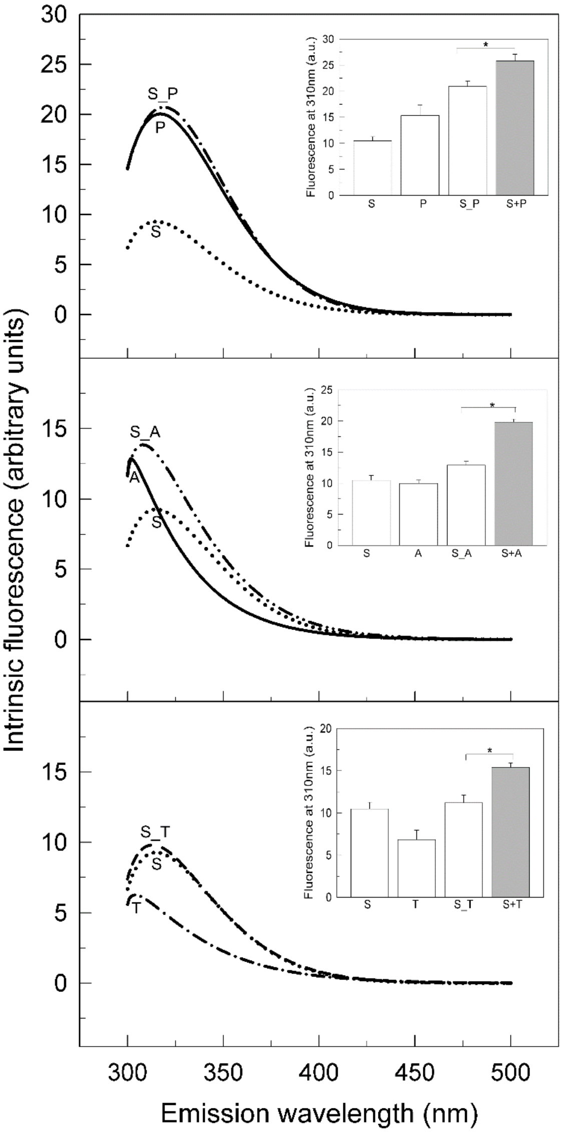

Figure S1. Steady-state emission spectra of the intrinsic fluorescence of sorcin, PMCA, Aβ, and tau. Spectra of 1 μM sorcin (S), 2.5 μg of PMCA (P), 30 μM of Aβ1-42 (A) and 300 nM tau (T) in 100 μL of fluorescence buffer were recorded separately and after incubating S with P (S-P) or A (S-A) or T (S-T). Inserts show the maximum signal of each spectrum (white bars) and their sum (S+P, S+A, S+T, grey bars). Data are mean ± SE from four different experiments (* $p \leq 0.001$).

Suplementary Methods

Determination of conformational changes by fluorescence spectroscopy

The intrinsic fluorescence of tyrosine residues in sorcin, PMCA, Aβ and tau was used to detect conformational changes due to their interactions. 1 μM sorcin, 2.5 μg PMCA, 30 μM of 2h aged Aβ and 300 nM tau were incubated separately or together in 100 μL of 150 mM PBS (pH 7.4), 100 mM KCl and 0.1 mM EDTA (fluorescence buffer), in Microfluor-1 black plates (384 wells). Fluorescence intensities were monitored in a Varioskan fluorescence spectrophotometer with the excitation wavelength set at 280 nm, and the emission spectra recorded between 300–500 nm.
